# Appendicitis

**Published:** 1904-11

**Authors:** 


### APPENDICITIS.

Robert T. Morris, when asked as to the method of preventing the disease says: "The removal of constipation is the removal of one of the dangers." There is constipation necessarily. It is due largely to interference with peristalsis. The history of the disease is a history of constipation. Then, when a person who is naturally more or less constipated, has the early, prodromic symptoms of appendicitis, let the constipation be corrected, and correction means restoration of natural peristalsis.

Let there be moderation in eating. Nourish him on easily digested diet. Get the bowels regular and keep them so. The free use of pure water is an effective laxative, obviating all drastic methods. Beware of purging. The small intestines do not need it. The colon is impacted, and its contents should be quickly washed down with a hot, saline, soap enema. With a little patience the water can cleanse the canal up to the cæcum; and the colon freed of its contents is an important beginning of treatment. Having cleansed the colon, follow by an enema of sweet oil, that the mucous membrane may be healed. Repeat the water if necessary.

Apply cold compresses to the affected abdominal area, to allay heat and pain. The clothing next to the skin should be light and porous. The relief of pain must be circumspect, or it will impair every chance of recovery, and set at naught every important feature of treatment. Do not give opium or morphine. When the disease has progressed so far as to demand operation, accede to the demand at once.—*Modern Medicine, May, 1904.*

